# Early prediction of severe retinopathy of prematurity requiring laser treatment using physiological data

**DOI:** 10.1038/s41390-023-02504-6

**Published:** 2023-02-14

**Authors:** Jarinda A. Poppe, Sean P. Fitzgibbon, H. Rob Taal, Sjoukje E. Loudon, Angela M. Tjiam, Charles C. Roehr, Irwin K. M. Reiss, Sinno H. P. Simons, Caroline Hartley

**Affiliations:** 1 Department of Neonatal and Pediatric Intensive Care, Division of Neonatology, Erasmus University Medical Center Sophia Children’s Hospital, Rotterdam, the Netherlands; 2 Wellcome Centre for Integrative Neuroimaging, FMRIB, Nuffield Department of Clinical Neurosciences, University of Oxford, Oxford, UK; 3 Department of Ophthalmology, Erasmus MC, University Medical Center Rotterdam, Rotterdam, the Netherlands; 4 National Perinatal Epidemiology Unit, Nuffield Department of Population Health, Medical Sciences, Division, University of Oxford, Oxford, UK; 5 Newborn Services, Southmead Hospital, North Bristol Trust, Bristol, UK; 6 Faculty of Health Sciences, University of Bristol, Bristol, UK; 7 Department of Paediatrics, University of Oxford, Oxford, UK

## Abstract

**Background:**

Early risk stratification for developing retinopathy of prematurity (ROP) is essential for tailoring screening strategies and preventing abnormal retinal development. This study aims to examine the ability of physiological data during the first postnatal month to distinguish preterm infants with and without ROP requiring laser treatment.

**Methods:**

In this cohort study, preterm infants with a gestational age <32 weeks and/or birth weight <1500 g, who were screened for ROP were included. Differences in the physiological data between the laser and non-laser group were identified, and tree-based classification models were trained and independently tested to predict ROP requiring laser treatment.

**Results:**

In total, 208 preterm infants were included in the analysis of whom 30 infants (14%) required laser treatment. Significant differences were identified in the level of hypoxia and hyperoxia, oxygen requirement, and skewness of heart rate. The best model had a balanced accuracy of 0.81 (0.72–0.87), a sensitivity of 0.73 (0.64–0.81), and a specificity of 0.88 (0.80–0.93) and included the SpO_2_/FiO_2_ ratio and baseline demographics (including gestational age and birth weight).

**Conclusions:**

Routinely monitored physiological data from preterm infants in the first postnatal month are already predictive of later development of ROP requiring laser treatment, although validation is required in larger cohorts.

**Impact:**

- Routinely monitored physiological data from the first postnatal month are predictive of later development of ROP requiring laser treatment, although model performance was not significantly better than baseline characteristics (gestational age, birth weight, sex, multiple birth, prenatal glucocorticosteroids, route of delivery, and Apgar scores) alone.
- A balanced accuracy of 0.81 (0.72–0.87), a sensitivity of 0.73 (0.64–0.81), and a specificity of 0.88 (0.80–0.93) was achieved with a model including the SpO_2_/FiO_2_ ratio and baseline characteristics.
- Physiological data have potential to play a significant role for future ROP prediction and provide opportunities for early interventions to protect infants from abnormal retinal development.

## Introduction

Retinopathy of prematurity (ROP) is a common disease in preterm infants, and accounts for 5–20% of childhood blindness in developed countries.^1^ The number of infants at risk of ROP has increased worldwide due to advances in perinatal and neonatal care with improved neonatal survival rates. Timely screening and intervention of high-risk infants for ROP is essential to prevent development of severe ROP.^2^ However, screening infants for ROP is thought to be painful, stressful and causes physiological instability.^3,4^ Classifying infants according to risk of developing ROP could be useful to identify those where ROP progression should be monitored carefully through extra screening and conversely avoid unnecessary screening examinations in infants who are predicted to be low risk. However, the American Academy of Ophthalmology recently assessed the accuracy of available prediction models for clinically significant ROP and reported that model optimization is needed before clinical application can be reached.^5^

Many putative risk factors for ROP have been described, including gestational age and birth weight, perinatal and postnatal inflammation, pulmonary complications, and anemia.^6^ Most guidelines use birth weight and gestational age to identify infants in need of ROP screening, which is highly sensitive, but results in screening of many infants who will not develop severe disease.^5^ Fluctuations in oxygenation, resulting in oxidative stress, are implicated in ROP.^7,8^ Several large randomized controlled trials reported a higher incidence of ROP when high oxygen saturation (SpO_2_) targets were used,^9,10^ although this association was not found in other trials.^11,12^ Hyperoxia related factors such as the use of supplemental oxygen, oxygen concentration, duration, and prolonged mechanical ventilation are among the most frequently described risk factors for the development of severe and treatment-requiring ROP.^6^

Most preterm infants in the neonatal intensive care unit are continuously monitored. Although the obtained physiological monitor data are used to detect trends and identify severe episodes of physiological instability, this snapshot method means that these data are often not used to their full potential and opportunities to improve clinical management may be missed.^13–15^ Analyzing physiological data could potentially lead to the identification of meaningful patterns that would otherwise be difficult or even impossible to identify, and may enable the prediction of individual risk for ROP. Previously Sullivan et al. investigated whether measures of heart rate and SpO_2_ in the first 7 days of life can improve predictions of mortality and morbidity in very low birth weight infants.^16^ Whilst physiological data improved prediction of death, severe intraventricular hemorrhage and bronchopulmonary dysplasia, it did not improve prediction of severe ROP beyond that made from demographics. A more detailed analysis of physiological data, including measures of oxygen requirement, may shed light on the predictive ability of these data for ROP and whether a particular timeframe has greater utility.

The aim of this study was to investigate if physiological monitor data and oxygen requirement during the first 30 days after birth—the period before the first ROP screening—differ between preterm infants with and without severe ROP requiring laser treatment, and to examine the ability of these data to predict infants who will develop severe ROP requiring laser treatment.

## Methods

### Study design and population

In this retrospective cohort study, physiological monitor data from the first postnatal month were analyzed and used to build classification models to distinguish infants with and without severe ROP requiring laser treatment. Preterm infants who were screened for ROP at the level III Neonatal Intensive Care Unit (NICU) of the Erasmus MC Sophia Children’s Hospital between August 2016 and August 2020 were eligible for inclusion. Exclusion criteria were a gestational age >32 weeks, a birth weight >1500 g, and <80% availability of the physiological data in the first two weeks after birth. Before the exclusion criteria were applied, the infants were randomly 1:1 divided using R Software (version 4.1.1, R Foundation for Statistical Computing, Vienna, Austria) into a training dataset and a test dataset. The medical ethics committee of the Erasmus University Medical Center granted a waiver from approval for this study according to the Dutch Medical Research Involving Human Subjects Act (MEC-2018–1106).

### Data acquisition

Demographic and clinical characteristics were collected from the electronic medical records. The DIGIROP-Birth was calculated using the website that the investigators of the score provided (https://www.digirop.com/index.html).^17^ Data on the fraction of inspired oxygen (FiO_2_), SpO_2_, and heart rate were collected from bedside monitors. Further details are given in the Supplementary Methods.

### ROP screening and outcome

The outcome of the study (i.e., the class for the machine learning) was whether infants did or did not develop severe ROP requiring laser coagulation. Laser coagulation was administered according to the ETROP criteria.^18^ The incidental use of anti-VEGF (bevacizumab) was not taken into account in this study. All preterm infants who were born with a gestational age <32 weeks and/or birth weight <1500 g were, according to local protocol, screened for ROP. The first screening was scheduled from 5 weeks postnatally, but not before 31 weeks of postmenstrual age. Data on ROP screenings were collected from the reports of the experienced ophthalmologists (S.E.L., A.M.T.) who performed the screening.

### Data processing

Features were derived from the data on the SpO_2_, heart rate, and FiO_2_ and calculated using the R Software (version 4.1.1, R Foundation for Statistical Computing, Vienna, Austria) or MATLAB (R2021a).^19^ The methods to calculate these features are presented in Supplemental Table 1. The skewness of the SpO_2_ and heart rate were calculated with the “skewness” function from the R package “e1071” as a measure of symmetry in the distribution of the data, with a value of 0 indicating that the data has a symmetric (normal) distribution.^20^ The mean SpO_2_, heart rate, FiO_2_, and SpO_2_/FiO_2_ ratio were calculated per day. The skewness of the SpO_2_ and heart rate, area under the 80% SpO_2_ curve, area above the 95% SpO_2_ curve, number of desaturations, number of bradycardia, and number of tachycardia were processed as total amount per day. Measurements marked as invalid by the bedside monitors were excluded from the analysis.

### Statistical analysis

Baseline characteristics were analyzed using the Wilcoxon rank sum test, *X*^2^ test, and Fisher’s exact test. Time periods with differences in each physiological feature between the laser and the non-laser group in the training data were identified using a non-parametric cluster analysis described by Maris and Oostenveld,^21^ see Supplementary Methods.

To explore the predictive ability of physiological data, random forest classification models were built on the training dataset to distinguish between the laser and the non-laser group, see Supplementary Methods. Model performance was measured using balanced accuracy, sensitivity, specificity and the Matthew’s correlation coefficient (MCC) with leave-one-out cross-validation in the training set. 95% confidence intervals were calculated with the Agresti–Coull interval.

To compare the predictive ability of physiological features at different time periods and with demographic features, 10 different models were built in the training dataset. Model 1 was trained on all physiological data from the first 30 days after birth. Model 2 was trained on the physiological features that contained significant differences between the laser and non-laser group, based on the non-parametric cluster analysis. Model 3 was trained on physiological features in a selected time period where most significant clusters were identified. To compare different physiological features, univariate analyses were performed including the physiological features from the first 30 days after birth; model 4 presents the best performing model from these analyses. After this, model 5 was trained on demographic features around birth only to compare the predictive ability of physiological data with demographic features. Then, the demographic features were added to the best performing models including physiological data (model 6 and 7). Model 8 included clinical features only (data on administered treatments and comorbidities; of note some of these occurred after the first 30 postnatal days) and model 9 and 10 also included the features of the best performing physiological models. As most infants who required laser treatment were born before 28 weeks of gestation a subgroup analysis was performed including infants born ≤28 weeks of gestation by retraining the two best performing models to increase the comparability between the laser and non-laser group.

Two-sided mid-*p* value McNemar’s tests^22^ were used to compare models. Significance was set to a *p* value <0.05; however, statistical comparisons were calculated as a guide only (without correction for multiple comparisons) and should not be interpreted as clearly indicating a better model as this is context dependent.^23^ The probability that an observation comes from a particular class was calculated by averaging over all trees in the ensemble using the MATLAB “predict” function. Differences in the sensitivity and specificity of the best performing models were calculated for different probability thresholds.

The two models with the highest performance scores were trained on the whole training set and externally validated by applying the models with the original probability threshold of 0.5 to the independent test set. These models were also trained on the training and test set combined to investigate if model performance improved by increasing sample size.

## Results

### Study population

A total of 269 preterm infants with a gestational age below 32 weeks were screened at least once for ROP. Overall, 61 (23%) infants were excluded from the analyses—34 in the training dataset and 27 in the test dataset. Infants were excluded due to a birth weight >1500 g (*N* = 9) or <80% availability of monitor data in the first 2 weeks after birth (*N* = 52, infants were admitted elsewhere first (*N* = 50) or due to technical problems (*N* = 2)). Of the remaining 208 infants, 100 were included in the training set and 108 in the independent test set. Thirty (14%) infants required laser treatment, of whom 15 infants were in the training dataset and 15 infants in the test dataset. The incidence of laser treatment did not differ between the training and the test dataset (*p* = 1.00), although the infants in the training dataset were more often male (*p* < 0.01) and had a longer NICU admission time (*p* = 0.04) (Supplemental Table 2).

### Demographic and clinical differences between the laser and non-laser group

Infants who required laser treatment (assessed in the training dataset) had a lower gestational age (*p* < 0.01) and birth weight (*p* < 0.01) compared to infants who did not require laser treatment (Table 1). Infants who required laser treatment more often received treatment with inotropes (*p* = 0.01) and dexamethasone (*p* < 0.01) during NICU admission. The DIGIROP probability was higher in the infants with laser treatment compared to the infants without laser treatment (*p* < 0.01).

**Table 1:** Baseline demographics of the population from the training dataset.

	All (*N* = 100)	No ROP requiring laser treatment (*N* = 85)	ROP requiring laser treatment (*N* = 15)	*p* value*
Gestational age (weeks)	26.4 (25.3–27.6)	26.6 (25.6–28.0)	25.0 (24.5–25.6)	<0.01
Birth weight (g)	830 (700–973)	855 (725–1020)	660 (577–790)	<0.01
SGA (yes)	19 (19%)	17 (20%)	2 (13%)	0.73
Sex (male)	69 (69%)	62 (73%)	7 (47%)	0.08
Multiple birth (yes)	24 (24%)	22 (26%)	2 (13%)	0.51
Mortality^a^ (yes)	4 (4%)	3 (4%)	1 (7%)	0.48
Apgar				
1 min	5 (3–7)	5 (3–7)	6 (5–7)	0.45
5 min	8 (7–8)	8 (7–8)	8 (7–9)	0.49
Route of delivery (section)	61 (61%)	50 (59%)	11 (73%)	0.39
Prenatal glucocorticoids (yes)	87 (87%)	75 (89%)	12 (80%)	0.41
Doses (n)	2 (1–2)	2 (1–2)	2 (1–2)	0.93
Treatments (yes)				
Surfactant	79 (79%)	65 (76%)	14 (93%)	0.18
Inotropes	35 (35%)	25 (29%)	10 (67%)	0.01
Caffeine	99 (99%)	84 (99%)	15 (100%)	1.00
Doxapram	49 (49%)	41 (48%)	8 (53%)	0.93
Dexamethasone	34 (34%)	22 (26%)	12 (80%)	<0.01
RBC transfusion	84 (84%)	69 (81%)	15 (100%)	0.12
iNO therapy	15 (15%)	10 (12%)	5 (33%)	0.08
Duration of NICU admission (days)	68 (53–94)	63 (50–82)	99 (86–108)	<0.01
Comorbidities (yes)				
NEC	13 (13%)	11 (13%)	2 (13%)	1.00
Sepsis	68 (68%)	55 (65%)	13 (87%)	0.13
IVH	19 (19%)	16 (19%)	3 (20%)	1.00
PDA	46 (46%)	38 (45%)	8 (53%)	0.74
DIGIROP probability (%)	6.1 (3.2–17.2)	5.2 (2.3–14.5)	17.8 (14.1–25.9)	<0.01

Data from the training set are presented as median (IQR) and *n* (%). *SGA* small for gestational age, *RBC* red blood cell, *iNO* inhaled nitric oxide, *NEC* necrotizing enterocolitis, *IVH* intraventricular hemorrhage, *PDA* patent ductus arteriosus.

*p value from the Wilcoxon rank sum test, *X*^2^ test, and Fisher’s exact test evaluated between the laser and non-laser group.

^a^The group without ROP requiring laser treatment had a postmenstrual age of 46–49 weeks at the time of death and ROP treatment was not expected anymore.

### Physiological data significantly differ between infants requiring laser treatment and those who do not

**Fig. 1:**
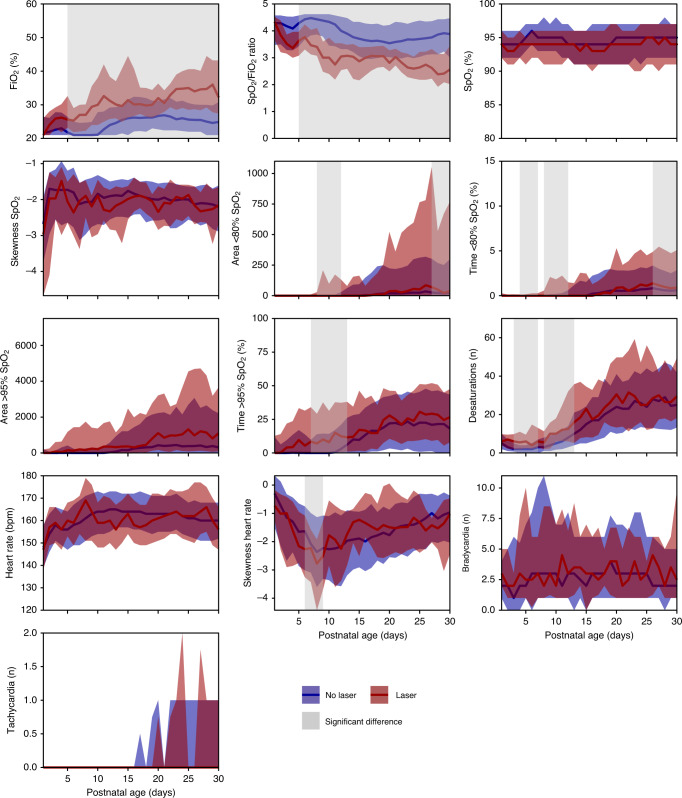
Graphs representing the physiological features for the group with and without laser treatment. Physiological features included the fraction of inspired oxygen (FiO_2_), oxygen saturation (SpO_2_)/FiO_2_ ratio, SpO_2_, skewness of the SpO_2_, area <80% SpO_2_ curve, time percentage <80% SpO_2_, area >95% SpO_2_ curve, time percentage >95% SpO_2_, incidence of desaturations, heart rate, skewness of the heart rate, and incidence of bradycardia and tachycardia in the first 30 postnatal days for the group with (red) and without (blue) laser treatment. Data are presented as median (IQR). The gray shaded areas mark time periods with significant differences (*p* < 0.05, non-parametric cluster analysis) between the laser and the non-laser groups.

In the training dataset, time periods were identified with a significantly higher FiO_2_ level, SpO_2_/FiO_2_ ratio, time spent at an SpO_2_ > 95%, and incidence of desaturations in the laser group compared to the non-laser group (Fig. 1). The area under the 80% SpO_2_, the percentage of time spent at an SpO_2_ ≤ 80%, and the skewness of the heart rate had significant time periods with lower levels in the laser group. No significant time periods were identified in the mean SpO_2_, the skewness in the SpO_2_, the area above the 95% SpO_2_, the mean heart rate, and the incidence of bradycardia and tachycardia.

### Physiological data are predictive of ROP treatment

**Fig. 2:**
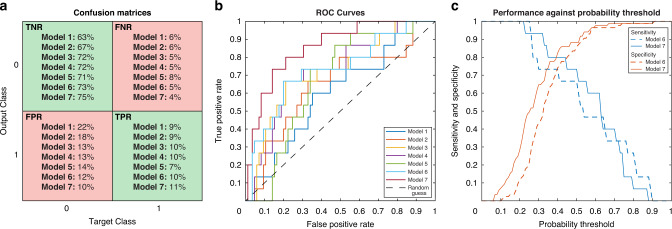
Graphs representing the performance of the classification models. **a** Confusion matrices of models including all physiological data from day 1 to day 30 after birth (model 1), including physiological data with significant clusters from day 1 to day 30 (model 2), including physiological data with significant clusters from day 5 to day 15 (model 3), including the SpO_2_/FiO_2_ ratio from day 1 to day 30 (model 4), including demographic features (model 5), including physiological data with significant clusters from day 5 to day 15 and demographic features (model 6), and including the SpO_2_/FiO_2_ ratio from day 1 to day 30 and demographic features (model 7). The green areas include the true positive and negative rates (TPR; TNR), and the red areas include the false positive and negative rates (FPR; FNR). Class 0 = no laser treatment, class 1 = laser treatment. **b** Receiving operating characteristics (ROC) curves of models 1–7 illustrating the discriminative ability of the models. A point at the diagonal dotted line means that it is not better than a random guess, a point above the dotted line means better than a random guess, and a point below the dotted line means worse than a random guess. **c** The sensitivity and specificity of the two best performing models (models 6 and 7) with a varying probability threshold set between 0 and 1.

A tree-based classification model including all physiological data from the first 30 postnatal days had a balanced accuracy of 0.67 (0.57–0.76), a sensitivity of 0.60 (0.50–0.69), and a specificity of 0.74 (0.65–0.82) in the training set (Table 2, model 1, and Fig. 2). Model performance was higher, though not significantly different, when including the physiological data with significant time periods only, based on the non-parametric cluster analysis, from the first 30 days (Table 2, model 2). The highest t statistic levels in most features were found between day 5–15 and day 25–30 (Supplemental Fig. 1). The balanced accuracy and specificity of model 3, included the physiological data with significant clusters from day 5 to day 15, were significantly higher compared to model 1 (Table 2). Adding the data from day 25 to day 30 to model 3 did not improve performance (Supplemental Table 3). Univariate analysis identified the SpO_2_/FiO_2_ ratio from the first 30 postnatal days as the best performing feature (Table 2, model 4; and Supplemental Table 3).

**Table 2:** Performance scores of the decision tree classification models, obtained in the training set.

Model	Features^a^	Balanced accuracy	Sensitivity	Specificity	MCC	*p* value*		
						Acc	Sens	Spec
Physiological features								
1	All physiological data (*day 1–30*)	0.67 (0.57–0.76)	0.60 (0.50–0.69)	0.74 (0.65–0.82)	0.26	N/A	N/A	N/A
2	Significant physiological data (*day 1–30*)	0.69 (0.60–0.78)	0.60 (0.50–0.69)	0.79 (0.70–0.86)	0.31	0.33^b^	1.00^b^	0.33^b^
3	Significant physiological data (*day 5–15*)	0.76 (0.66–0.83)	0.67 (0.57–0.75)	0.85 (0.76–0.91)	0.44	0.01^b^	0.50^b^	0.02^b^
4	SpO_2_/FiO_2_ ratio (*day 1–30*)	0.76 (0.66–0.83)	0.67 (0.57–0.75)	0.85 (0.76–0.91)	0.44	0.03^b^	0.63^b^	0.03^b^
Demographic features								
5	Demographics	0.65 (0.55–0.74)	0.47 (0.37–0.56)	0.84 (0.75–0.90)	0.26	0.44^c^ 0.47^d^	0.22^c^ 0.29^d^	0.83^c^ 0.84^d^
Demographic and physiological features								
6	Model 3 + demographics	0.76 (0.67–0.84)	0.67 (0.57–0.75)	0.86 (0.78–0.92)	0.45	0.31^e^	0.22^e^	0.65^e^
7	Model 4 + demographics	0.81 (0.72–0.87)	0.73 (0.64–0.81)	0.88 (0.80–0.93)	0.54	0.08^e^	0.13^e^	0.30^e^
Clinical features								
8	Clinical data	0.73 (0.63–0.81)	0.60 (0.50–0.69)	0.86 (0.78–0.92)	0.40	0.18^c,d^	0.73^c,d^	0.82^c,d^
Demographic, clinical, and physiological features								
9	Model 6 + clinical data	0.74 (0.64–0.81)	0.60 (0.50–0.69)	0.87 (0.79–0.92)	0.42	0.17^f^	0.50^f^	0.69^f^
10	Model 7 + clinical data	0.84 (0.75–0.90)	0.73 (0.64–0.81)	0.94 (0.87–0.98)	0.66	0.07^g^	0.27^g^	0.07^g^

The model performance is reported as the balanced accuracy, sensitivity, specificity, and Matthew’s correlations coefficient (MCC) with the 95% CI for the balanced accuracy, sensitivity, and specificity.

*p value calculated by the McNemar’s test.

^a^Physiological data included data on SpO_2_, skewness SpO_2_, incidence of desaturations, area under the 80% SpO_2_ curve, area above the 95% SpO_2_ curve, percentage of time below 80% SpO_2_, percentage of time above 95% SpO_2_, FiO_2_, SpO_2_/FiO_2_ ratio, heart rate, incidence of bradycardia, incidence of tachycardia, skewness heart rate. Demographics included data on gestational age, birth weight, sex, multiple birth, number of prenatal glucocorticosteroids doses, route of delivery, and Apgar score at 1 and 5 min. Clinical data included data during NICU admission on treatment with surfactant, inotropes, doxapram, dexamethasone, red blood cell transfusions, and nitric oxide, and data on the occurrence of necrotizing enterocolitis, sepsis, intraventricular hemorrhage, and patent ductus arteriosus.

^b^Compared to model 1.

^c^Compared to model 3.

^d^Compared to model 4.

^e^Compared to model 5.

^f^Compared to model 6.

^g^Compared to model 7.

### Combining physiological with demographic and clinical features

The balanced accuracy, sensitivity, and specificity of the model including demographic features (gestational age, birth weight, sex, multiple birth, amount of prenatal glucocorticosteroids doses, route of delivery, and Apgar score at 1 and 5 min) only (Table 2, model 5) were lower, although not significantly, compared to models 3 and 4 with physiological data only. We next investigated whether combining demographic and physiological data improved prediction. Adding the demographics to the model including the incidence of desaturations, area under the 80% SpO_2_ curve, percentage of time below 80% SpO_2_, percentage of time above 95% SpO_2_, FiO_2_, SpO_2_/FiO_2_ ratio, and skewness heart rate from day 5 to day 15, did not significantly improve model performance (Table 2, model 6). Combining the SpO_2_/FiO_2_ ratio from day 1 to day 30 and the demographics resulted in a higher performance, although this was not significantly different (Table 2, model 7). Model 6 had a positive predictive value of 0.45 and a negative predictive value of 0.94, and model 7 had a positive and negative predictive value of 0.52 and 0.95 respectively. Adding clinical data during NICU admission on treatment and comorbidities did not significantly improve model performance (Table 2, models 8–10).

As most infants who required laser treatment were born before 28 weeks’ gestation models may be particularly applicable in the youngest infants. The subgroup analysis including infants with a gestational age ≤28 weeks only (*N* = 79) was performed by retraining model 6 and 7 as they were the highest performing models using data collected in the first 30 postnatal days before ROP screening is conducted (unlike the models including clinical data). Model 6 applied to infants in the subgroup had a balanced accuracy of 0.69 (0.58–0.78), a sensitivity of 0.60 (0.49–0.70), a specificity of 0.78 (0.68–0.86) and a MCC of 0.33. Model 7 applied to infants in the subgroup had a balanced accuracy of 0.77 (0.67–0.85), a sensitivity of 0.67 (0.56–0.76), a specificity of 0.88 (0.78–0.93) and a MCC of 0.51.

### Improved identification of infants requiring laser treatment and model validation

To determine whether a model can identify all infants requiring laser treatment, the probability thresholds of the best performing models were varied (Fig. 2c). With a probability threshold of 0.25 in model 6 and 0.22 in model 7 15/15 infants requiring laser treatment could be identified (sensitivity = 1.00) at the expense of 67/85 false positive infants (specificity = 0.21) in model 6 and 52/85 false positive infants (specificity = 0.39) in model 7; this would still enable a substantial reduction in the number of infants unnecessarily screened.

Models 6 and 7 were validated on the data of the independent test set (Table 3). To assess whether sample size had an effect on performance the models were also retrained on data from the training and test set combined (Table 3).

**Table 3:** Performance scores of the validated classification models including data from the test set and data of the training and test set combined.

Validated model	Dataset	Balanced accuracy	Sensitivity	Specificity	MCC
6	Test dataset	0.66 (0.57–0.75)	0.67 (0.57–0.75)	0.66 (0.56–0.74)	0.23
6	Test and training dataset	0.77 (0.71–0.82)	0.67 (0.60–0.73)	0.87 (0.82–0.91)	0.47
7	Test dataset	0.68 (0.59–0.77)	0.53 (0.44–0.63)	0.84 (0.75–0.89)	0.31
7	Test and training dataset	0.79 (0.73–0.84)	0.67 (0.60–0.73)	0.91 (0.86–0.94)	0.53

The model performance is reported as the balanced accuracy, sensitivity, specificity, and Matthew’s correlations coefficient (MCC) with the 95% CI for the balanced accuracy, sensitivity, and specificity.

## Discussion

This study investigated whether routinely monitored physiological data from the first 30 postnatal days (a clinically relevant period before the first ROP screening) could be used for the early identification of preterm infants at risk of severe ROP requiring laser treatment. Differences were observed in the level of hypoxia and hyperoxia, the fractional inspired oxygen requirement, and skewness of the heart rate; and the physiological data, especially the SpO_2_/FiO_2_ ratio, could be used to achieve good predictions of ROP requiring laser treatment. Prediction performance was increased, though not significantly, by including baseline demographics and clinical factors. Importantly, good predictions were achieved using physiological data from only day 5 to day 15 after birth, highlighting both the early predictive ability of physiological data and a time window during which infants may be especially vulnerable to oxidative stress and so could particularly benefit from careful management of oxygenation and ventilation.

Periods with increased use of supplemental oxygen and a higher level of hypoxia and hyperoxia were detected in the first postnatal weeks in infants with ROP requiring laser treatment. The association between ROP and the use of supplemental oxygen, periods of hyperoxia and hypoxia and time outside the targeted saturation range have been described in multiple studies.^2,12,24–26^ The classic oxygen-induced retinopathy animal model describes the effect of hypoxia and hyperoxia in the pathophysiology of ROP with vaso-obliteration during hyperoxia exposure and vaso-proliferation during relative hypoxia, although further research is still needed for detailed understanding of pathological changes.^27^ The ability of physiological and demographic data to predict severe ROP has been previously investigated by Sullivan et al.^16^, although the pulse oximetry variables did not improve prediction in their analysis. Adding physiological data to the demographic data in our study did not significantly increase the performance of the models. However, 4 more patients out of 15 were correctly identified as high-risk patients for developing ROP compared with the model using demographic data only which is clinically important and the lack of statistical significance is likely limited by the small sample size. The use of supplemental oxygen, and consequently the SpO_2_/FiO_2_ ratio, was higher in our study during the whole study period apart from the first few days after birth. The SpO_2_/FiO_2_ ratio performed best in the univariate analyses and might be a sufficient reflection of the saturation profiles of patients at risk.

Recently, the DIGIROP-Birth and DIGIROP-Screen have been developed to predict ROP requiring treatment using birth demographics and ROP progression data.^17,28^ The DIGIROP-Birth probability in our data was significantly higher in the laser group compared to the non-laser group. In initial model development we aimed to achieve high accuracy; however, clinically it is essential that no infants requiring treatment are missed. Varying the probability threshold of our model enabled us to achieve 100% sensitivity, with a specificity of 39%, similar to the results of DIGIROP-Screen at birth, and similar or higher compared to former developed models including the WINROP, G-ROP criteria, and a model based on Swiss data.^28–31^ As physiological data from only the first 30 days after birth were included in our model, the number of infants who are unnecessarily screened could already be reduced by 40% prior to the first screening. We speculate that combining the physiological data with the ROP progression data could further improve prediction, especially when using photographic documentation and telemedicine.^32,33^

A limitation of our study is the relatively low number of infants with ROP requiring laser treatment and the small overall data size. This likely explains the drop in performance of the classification models when applied to the independent test cohort. Intra- and inter-site differences should be assessed to further optimize the algorithm, using much larger sample sizes.^34^ This highlights the need for sharing physiological and neonatal outcome data; developing a consensus of a standardized format for data sharing is important to facilitate these aims.^13^ The NEDROP2 study and a large Swiss study reported that, respectively, 39 out of 1085 (3.6%) and 94 out of 7817 (1.2%) fully screened infants received treatment for developing ROP.^35,36^ This is lower compared to the 14% of infants in our study who required laser treatment, probably because our study population is not fully comparable to these studies which included infants born at both high-care and regional centers and highlights the likely need to include infants from lower-care settings to create a model that will be accurate in all centers. Moreover, the relatively small sample size may have exacerbated problems caused by imbalance of the data. Training decision tree classifiers on an unbalanced dataset can lead to frequency bias and a poor accuracy, by learning from data observations that occur more frequently. To avoid bias and increase the model performance we used a random undersampling algorithm, and balanced accuracy was calculated to better reflect model performance.

In summary, routinely monitored physiological data is predictive of ROP requiring laser treatment in preterm infants, although it did not significantly improve prediction from baseline characteristics in this study. Continuous analysis of these data will improve our understanding of important time periods when infants are at risk for developing ROP to optimize preventive policies. Combining physiological data with existing risk scores and automatic photographic documentation needs further investigation with larger sample sizes but could potentially enhance objective assessment of ROP risk profiles in the future.

## Supplementary Information

Supplementary methods

## Data Availability

All data generated or analyzed during this study are included in this article and can be obtained from the corresponding author upon request.

## References

[1] Gilbert C (2008). Retinopathy of prematurity: a global perspective of the epidemics, population of babies at risk and implications for control. Early Hum. Dev..

[2] Gantz MG (2020). Achieved oxygen saturations and retinopathy of prematurity in extreme preterms. Arch. Dis. Child. Fetal Neonatal Ed..

[3] Mitchell AJ, Green A, Jeffs DA, Roberson PK (2011). Physiologic effects of retinopathy of prematurity screening examinations. Adv. Neonatal Care.

[4] Belda S, Pallas CR, De la Cruz J, Tejada P (2004). Screening for retinopathy of prematurity: is it painful?. Biol. Neonate.

[5] Hutchinson AK (2016). Clinical models and algorithms for the prediction of retinopathy of prematurity: a Report by the American Academy of Ophthalmology. Ophthalmology.

[6] Kim SJ (2018). Retinopathy of prematurity: a review of risk factors and their clinical significance. Surv. Ophthalmol..

[7] Kermorvant-Duchemin E (2005). Trans-arachidonic acids generated during nitrative stress induce a thrombospondin-1-dependent microvascular degeneration. Nat. Med..

[8] Garg U (2012). Free radical status in retinopathy of prematurity. Indian J. Clin. Biochem..

[9] Carlo WA (2010). Target ranges of oxygen saturation in extremely preterm infants. N. Engl. J. Med..

[10] Stenson BJ (2013). Oxygen saturation and outcomes in preterm infants. N. Engl. J. Med..

[11] Schmidt B (2013). Effects of targeting higher vs lower arterial oxygen saturations on death or disability in extremely preterm infants: a randomized clinical trial. JAMA.

[12] Supplemental Therapeutic Oxygen for Prethreshold Retinopathy of Prematurity (STOP-ROP), a randomized, controlled trial. I: Primary outcomes. *Pediatrics* **105**, 295–310 (2000). 10.1542/peds.105.2.295 10654946

[13] Hartley C (2021). Toward personalized medicine for pharmacological interventions in neonates using vital signs. Paediatr. Neonatal Pain.

[14] Poppe JA (2020). Precision dosing of doxapram in preterm infants using continuous pharmacodynamic data and model-based pharmacokinetics: an illustrative case series. Front. Pharmacol..

[15] Poppe JA (2020). Use of continuous physiological monitor data to evaluate doxapram therapy in preterm infants. Neonatology.

[16] Sullivan BA (2018). Early pulse oximetry data improves prediction of death and adverse outcomes in a two-center cohort of very low birth weight infants. Am. J. Perinatol..

[17] Pivodic A (2020). Individual risk prediction for sight-threatening retinopathy of prematurity using birth characteristics. JAMA Ophthalmol..

[18] Good WV, Early Treatment for Retinopathy of Prematurity Cooperative Group. (2004). Final results of the Early Treatment for Retinopathy of Prematurity (ETROP) randomized trial. Trans. Am. Ophthalmol. Soc..

[19] Hartley C (2018). Analgesic efficacy and safety of morphine in the Procedural Pain in Premature Infants (Poppi) Study: randomised placebo-controlled trial. Lancet.

[20] Meyer, D. et al. E1071: misc functions of the Department of Statistics, Probability Theory Group (Formerly: E1071), Tu Wien [R Package Version 1.7-9]. https://CRAN.R-project.org/package=e1071 (2021).

[21] Maris E, Oostenveld R (2007). Nonparametric statistical testing of EEG- and MEG-Data. J. Neurosci. Methods.

[22] Fagerland MW, Lydersen S, Laake P (2013). The Mcnemar test for binary matched-pairs data: mid-P and asymptotic are better than exact conditional. BMC Med. Res. Methodol..

[23] van der Vaart M (2019). Multimodal pain assessment improves discrimination between noxious and non-noxious stimuli in infants. Paediatr. Neonatal Pain.

[24] Di Fiore JM (2012). The relationship between patterns of intermittent hypoxia and retinopathy of prematurity in preterm infants. Pediatr. Res..

[25] Vesoulis ZA, Lust CE, Liao SM, Trivedi SB, Mathur AM (2016). Early hyperoxia burden detected by cerebral near-infrared spectroscopy is superior to pulse oximetry for prediction of severe retinopathy of prematurity. J. Perinatol..

[26] Srivatsa, B., Hagan, J. L., Clark, R. H. & Kupke, K. G. Oxygenation factors associated with retinopathy of prematurity in infants of extremely low birth weight. *J. Pediatr*. **247**, 46.e4–52.e4 (2022). 10.1016/j.jpeds.2022.03.057 35427689

[27] Tedeschi T, Lee K, Zhu W, Fawzi AA (2022). Limited hyperoxia-induced proliferative retinopathy: a model of persistent retinal vascular dysfunction, preretinal fibrosis and hyaloidal vascular reprogramming for retinal rescue. PLoS ONE.

[28] Pivodic, A. et al. Development and validation of a new clinical decision support tool to optimize screening for retinopathy of prematurity. *Br. J. Ophthalmol*. **106**, 1573–1580 (2021). 10.1136/bjophthalmol-2020-318719 PMC8627649 33980506

[29] Binenbaum G (2018). Development of modified screening criteria for retinopathy of prematurity: primary results from the postnatal growth and retinopathy of prematurity study. JAMA Ophthalmol..

[30] Gerull R (2018). Prediction of ROP treatment and evaluation of screening criteria in vlbw infants-a population based analysis. Pediatr. Res..

[31] Lundgren P (2013). Winrop identifies severe retinopathy of prematurity at an early stage in a nation-based cohort of extremely preterm infants. PLoS ONE.

[32] Valikodath N, Cole E, Chiang MF, Campbell JP, Chan RVP (2019). Imaging in retinopathy of prematurity. Asia Pac. J. Ophthalmol..

[33] Wu Q (2022). Development and validation of a deep learning model to predict the occurrence and severity of retinopathy of prematurity. JAMA Netw. Open.

[34] Zimmet AM (2021). Vital sign metrics of VLBW infants in three nicus: implications for predictive algorithms. Pediatr. Res..

[35] Trzcionkowska, K., Termote, J. U., Bohringer, S., van Sorge, A. J. & Schalij-Delfos, N. Nationwide inventory on retinopathy of prematurity screening in the Netherlands. *Br. J. Ophthalmol*. 10.1136/bjophthalmol-2021-319929 (2021). 10.1136/bjophthalmol-2021-319929 PMC10176329 34893474

[36] Gerull R (2018). Incidence of retinopathy of prematurity (ROP) and ROP treatment in Switzerland 2006-2015: a population-based analysis. Arch. Dis. Child. Fetal Neonatal Ed..

