# Supplementary material for: Early prediction of severe retinopathy of prematurity requiring laser treatment using physiological data
Source: Pediatr Res. 2023 Feb 14;94(2):699–706. doi: 10.1038/s41390-023-02504-6 (PMC10382319; doi:10.1038/s41390-023-02504-6)
Supplement: Supplementary file 1 — Supplementary methods [file 41390_2023_2504_MOESM1_ESM.pdf]

Supplementary methods

Data acquisition

Baseline characteristics, including gestational age, birth weight, sex, multiple birth, Apgar scores, route of delivery, and the use of maternal prenatal glucocorticosteriods, were collected from electronic medical records (HiX version 6.1, Chipsoft, Amsterdam, Netherlands). Administered treatments, including surfactant, inotropes, caffeine, doxapram, dexamethasone, red blood cell transfusion, and inhaled nitric oxide; and comorbidities including sepsis, necrotizing enterocolitis, intraventricular hemorrhage, and patent ductus arteriosus during NICU admission were also collected from the electronic medical records. Small for gestational age was calculated according to Fenton and Kim [1]. The DIGIROP-Birth, an early estimating risk model for ROP treatment using birth characteristics, was calculated using the website that the investigators of the score provided (https://www.digirop.com/index.html) [2]. Data on ROP screenings were collected from the reports of the experienced ophthalmologists (SL, AT) who performed the screening. Data on the fraction of inspired oxygen (FiO_2) were collected from the electronic patient data management system (Picis Clinical Solutions, Inc., Wakefield, MA, USA) and HiX. Only changes in the FiO_2 settings were recorded before 2017, after this the FiO_2 was continuously recorded each minute. Continuously logged SpO_2 and heart rate (sampled at 1Hz) were automatically collected from bedside monitors (Draegerwerk AG & Co KGaA, Lübeck, Germany). Data were captured from admittance until 30 days after birth, before the first ROP screening took place.

Statistical analysis

Non-parametric cluster analysis

Time periods with differences in each physiological feature between the laser and the non-laser group in the training data were identified using a non-parametric cluster analysis described by Maris and Oostenveld [3]. Briefly, differences were identified by calculating a t statistic at each day for each physiological feature as a measure of statistical difference between the two groups. Clusters were defined as contiguous sections of data with a t statistic above threshold set as 97.5 percentile of the t distribution; the start of a significant cluster was defined as the earliest time point with a t statistic above the threshold and the end of a cluster was defined as the time point with a t statistic below the threshold. Significant clusters were identified by comparison of the clusters attained from 1000 random permutations of the data indicating significant differences in the physiological data that occur for longer duration than would be expected by chance.

Machine learning analysis

The model training and statistical analysis were conducted using the ‘fitcensemble’ function in MATLAB (R2021a). Missing data were assumed to be completely at random and a model approach was chosen that is modifiable to account for missing data. The classification models were created with the random undersampling boosting (RUSBoost) algorithm to take into account class imbalance due to the relatively low incidence of ROP requiring laser treatment [4]. The RUSBoost algorithm is found to outperform other methods when training a classifier on unbalanced datasets [4]. The algorithm undersamples the class containing the most infants by taking only the number of observations of the class with the fewest infants (RatioToSmallest [1,1]).

References

1. Fenton TR, Kim JH. A systematic review and meta-analysis to revise the Fenton growth chart for preterm infants. *BMC Pediatr* 2013;13:59.
2. Pivodic A, Hard AL, Lofqvist C, Smith LEH, Wu C, Brunder MC, et al. Individual Risk Prediction for Sight-Threatening Retinopathy of Prematurity Using Birth Characteristics. *JAMA Ophthalmol* 2020;138(1):21-9.
3. Maris E, Oostenveld R. Nonparametric statistical testing of EEG- and MEG-data. *J Neurosci Methods* 2007;164(1):177-90.
4. Seiffert C, Khoshgoftaar TM, Van Hulse J, Napolitano A. RUSBoost: A Hybrid Approach to Alleviating Class Imbalance. *IEEE* 2010;40(1):185-97.

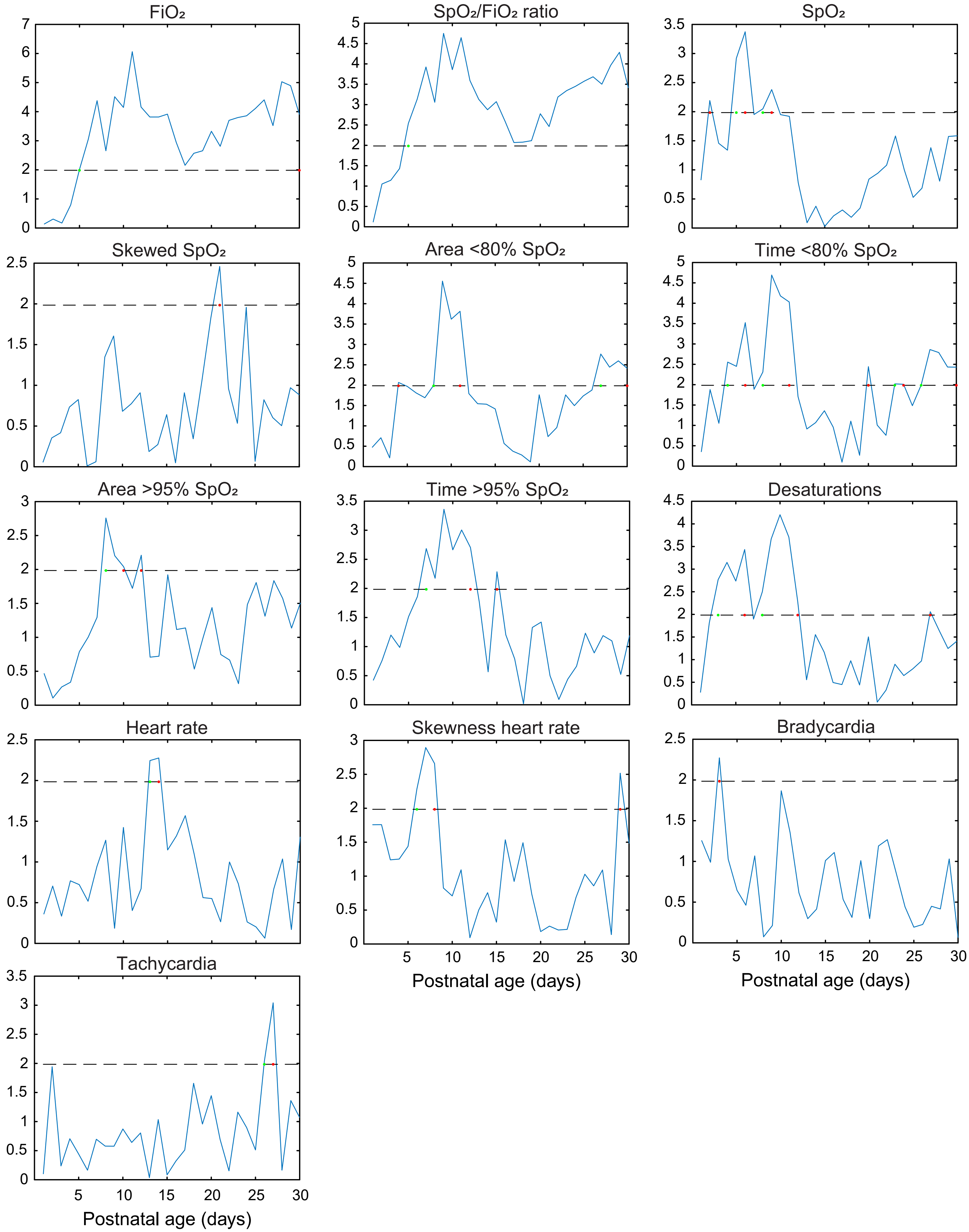

Supplemental Figure 1. Graphs presenting the t statistic between the group with and without laser treatment of the fraction of inspired oxygen (FiO_2), oxygen saturation (SpO_2)/FiO_2 ratio, SpO_2, skewness of the SpO_2, area <80% SpO_2 curve, time percentage <80% SpO_2, area >95% SpO_2 curve, time percentage >95% SpO_2, incidence of desaturations, heart rate, skewness of the heart rate, and incidence of bradycardia and tachycardia in the first 30 postnatal days. The dotted line marks the threshold set as 97.5 percentile of the t distribution.

Supplemental Table 1. Calculation of the derived features from the oxygen saturation, heart rate and fraction of inspired oxygen as source data.

**Source data**	**Feature**	**Calculation**
SpO_2	Skewness SpO_2	Using 'skewness' function R package 'e1071'
SpO_2	Incidence of desaturations	Episodes of desaturation were identified as periods during which SpO_2 levels decreased below 80% for at least 10 seconds.
SpO_2	Area under the 80% SpO_2 curve	Multiplying the difference between the SpO_2 limit of 80% and the measured SpO_2 by the time spent below the SpO_2 limit of 80%.
SpO_2, FiO_2	Area above the 95% SpO_2 curve	Multiplying the difference between the SpO_2 limit of 95% and the measured SpO_2 by the time spent above the SpO_2 limit of 95%. SpO_2 values >95% were not taken into account when FiO_2 was ≤21%.
SpO_2	Percentage of time below 80% SpO_2	Dividing the number of measurements below the SpO_2 limit of 80% by the available number of measurements per day.
SpO_2, FiO_2	Percentage of time above 95% SpO_2	Dividing the number of measurements above the SpO_2 limit of 95% by the available number of measurements per day. SpO_2 values >95% were not taken into account when FiO_2 was ≤21%.
SpO_2, FiO_2	SpO_2/FiO_2 ratio	Calculated per second where both SpO_2 and FiO_2 measurements were available.
Heart rate	Incidence of bradycardia	Episodes of bradycardia were defined as periods during which the heart rate decreased below 100 beats per minute for at least 15 seconds.
Heart rate	Incidence of tachycardia	Episodes of tachycardia were defined as periods during which the heart rate was greater than 200 beats per minute for at least 15 seconds.
Heart rate	Skewness heart rate	Using 'skewness' function R package 'e1071'

Supplemental Table 2. Baseline characteristics from the training and test set.

	**Training set (N=100)**	**Test set (N=106)**	***P*-value***
**ROP requiring laser** (yes)	15 (15%)	15 (14%)	1.00
**Gestational age** (weeks)	26.4 (25.3-27.6)	26.4 (25.6-27.3)	0.88
**Birth weight** (grams)	830 (700-973)	830 (686-989)	0.90
**SGA** (yes)	19 (19%)	18 (17%)	0.84
**Male** (yes)	69 (69%)	49 (46%)	<0.01
**Multiple birth** (yes)	24 (24%)	21 (20%)	0.58
**Mortality** (yes)	4 (4%)	7 (7%)	0.54
**Apgar**			
1 min	5 (3-7)	5 (4-7)	0.29
5 min	8 (7-8)	8 (7-9)	0.68
**Section** (yes)	61 (61%)	68 (64%)	0.75
**Prenatal glucocorticoids** (yes)	87 (87%)	93 (88%)	1.00
Doses (n)	2 (1-2)	2 (2-2)	0.23
**Treatments** (yes)			
Surfactant	79 (79%)	83 (78%)	1.00
Inotropes	35 (35%)	30 (28%)	0.38
Caffeine	99 (99%)	106 (100%)	0.49
Doxapram	49 (49%)	47 (44%)	0.60
Dexamethasone	34 (34%)	35 (33%)	1.00
RBC transfusion	84 (84%)	79 (75%)	0.13
iNO therapy	15 (15%)	15 (14%)	1.00
**Duration of NICU admission**	68 (53-94)	59 (47-80)	0.04
**Comorbidities** (yes)			
NEC	13 (13%)	16 (15%)	0.82
Sepsis	68 (68%)	68 (64%)	0.66
IVH	19 (19%)	28 (26%)	0.27
PDA	46 (46%)	43 (41%)	0.52

Data from the training and test set are presented as median (IQR) and n (%). SGA: small for gestational age, RBC: red blood cell, iNO: inhaled nitric oxide, NEC: necrotizing enterocolitis, IVH: intraventricular hemorrhage, PDA: patent ductus arteriosus.

**P*-value from the Wilcoxon rank sum test, X^2 test, and Fisher's exact test evaluated between the training and test set.

Supplemental Table 3. Performance scores of the decision tree classification models, obtained in the training set.

**Features***	**Balanced accuracy**	**Sensitivity**	**Specificity**	**MCC**	**P-value****		
					Acc	*Sens*	*Spec*
**Day 1-30**							
SpO_2/FiO_2 ratio	0.76 (0.66-0.83)	0.67 (0.57-0.75)	0.85 (0.76-0.91)	0.44	0.03[a]	0.63[a]	0.03[a]
Incidence of desaturations	0.67 (0.58-0.76)	0.53 (0.44-0.63)	0.81 (0.72-0.88)	0.29	0.33[a]	0.69[a]	0.19[a]
Area <80% SpO_2 curve	0.60 (0.50-0.69)	0.53 (0.44-0.63)	0.66 (0.56-0.74)	0.14	0.21[a]	0.69[a]	0.24[a]
Percentage of time <80% SpO_2	0.68 (0.59-0.77)	0.60 (0.50-0.69)	0.76 (0.67-0.84)	0.29	0.70[a]	1.00[a]	0.68[a]
Percentage of time >95% SpO_2	0.55 (0.45-0.64)	0.47 (0.37-0.56)	0.64 (0.54-0.72)	0.08	0.07[a]	0.45[a]	0.10[a]
FiO_2	0.68 (0.59-0.77)	0.53 (0.44-0.63)	0.84 (0.75-0.90)	0.32	0.13[a]	0.63[a]	0.06[a]
Skewness heart rate	0.54 (0.44-0.64)	0.40 (0.31-0.50)	0.68 (0.59-0.77)	0.06	0.15[a]	0.13[a]	0.34[a]
**Day 5-15**							
All physiological data	0.75 (0.66-0.83)	0.67 (0.57-0.75)	0.84 (0.75-0.90)	0.42	0.02[a]	0.50[a]	0.04[a]
SpO_2/FiO_2 ratio	0.66 (0.56-0.74)	0.47 (0.37-0.56)	0.85 (0.76-0.91)	0.28	0.15[a]	0.38[a]	0.04[a]
Incidence of desaturations	0.60 (0.50-0.69)	0.47 (0.37-0.56)	0.73 (0.63-0.81)	0.15	0.52[a]	0.38[a]	0.81[a]
Area <80% SpO_2 curve	0.68 (0.58-0.76)	0.60 (0.50-0.69)	0.75 (0.66-0.83)	0.28	0.86[a]	0.40[a]	0.85[a]
Percentage of time <80% SpO_2	0.71 (0.61-0.79)	0.60 (0.50-0.69)	0.81 (0.72-0.88)	0.34	0.23[a]	1.00[a]	0.21[a]
Percentage of time >95% SpO_2	0.63 (0.53-0.71)	0.53 (0.44-0.63)	0.72 (0.62-0.80)	0.19	0.60[a]	0.75[a]	0.68[a]
FiO_2	0.67 (0.57-0.75)	0.47 (0.37-0.56)	0.87 (0.79-0.92)	0.31	0.03[a]	0.38[a]	<0.01[a]
Skewness heart rate	0.58 (0.48-0.67)	0.53 (0.44-0.63)	0.62 (0.53-0.71)	0.11	0.08[a]	0.63[a]	0.10[a]
**Day 5-15, 25-30**							
Significant physiological data	0.70 (0.60-0.78)	0.53 (0.44-0.63)	0.86 (0.78-0.92)	0.35	0.77[b]	0.25[b]	0.75[b]

The model performance is reported as the balanced accuracy, sensitivity, specificity, and Matthew's correlations coefficient (MCC) with the 95% CI for the balanced accuracy, sensitivity, and specificity.

*****Physiological data included data on SpO_2, skewness SpO_2, incidence of desaturations, area under the 80% SpO_2 curve, area above the 95% SpO_2 curve, percentage of time below 80% SpO_2, percentage of time above 95% SpO_2, FiO_2, SpO_2/FiO_2 ratio, heart rate, incidence of bradycardia, incidence of tachycardia, skewness heart rate.

** P-value calculated by the McNemar's test.

[a]Compared to model 1

[b]Compared to model 3
